# From Synthesis to Sensing: The Insight into the Properties of Fe_3_O_4_ Magnetic Nanoparticles and Their Surface Modification Strategies in Voltammetric Trace Determination of Heavy Metal Ions

**DOI:** 10.3390/molecules30183796

**Published:** 2025-09-18

**Authors:** Damian Gorylewski, Katarzyna Tyszczuk-Rotko

**Affiliations:** Faculty of Chemistry, Institute of Chemical Sciences, Maria Curie-Skłodowska University in Lublin, 20-031 Lublin, Poland; damian.gorylewski@mail.umcs.pl

**Keywords:** heavy metal ions, Fe_3_O_4_ magnetic nanoparticles, voltammetry, electrode materials

## Abstract

Magnetic nanoparticles (MNPs) of iron oxide are becoming increasingly popular due to their excellent physicochemical properties as well as very good adsorption and catalytic properties towards heavy metal ions (HMIs). They are used in many industries and are becoming a desirable electrode material in voltammetry. Unfortunately, they tend to aggregate and easily oxidize. To mitigate these issues, they are often coated with organic or inorganic materials, which reduce oxidation and aggregation, and introduce an additional number of active sites capable of interacting with the analyte. Another approach involves the use of carbon material as a base for nanoparticles, which also improves the parameters of nanoparticles. This review reveals a condensed concept presenting functionalized Fe_3_O_4_ magnetic nanoparticles from the methods of their synthesis and modification to their application in the voltammetric trace analysis of HMIs. This paper describes the effect of electrode surface modification strategies on the stability of MNPs and the homogeneity of their distribution on the carbonaceous carrier surface. The authors focused on the voltammetric procedures for the single and simultaneous determination of HMIs using different electrode materials modified with Fe_3_O_4_ magnetic nanoparticles.

## 1. Introduction

The intensive development of new technologies is associated with the increased activity of heavy industry and the production of huge amounts of waste. Rapid industrialization in developing countries, particularly within sectors such as metal plating, ore extraction, battery manufacturing, fertilizer production, leather processing, and pharmaceutical synthesis, is leading to an increased burden of heavy metal ions (HMIs) in water systems, harming the natural environment [1,2,3]. The historical conceptualization of HMIs emerged in the early twentieth century, with density serving as the primary defining characteristic. In 1936 Niels Bjerrum proposed a classification based on densities of heavy metals exceeding 7 g cm^−3^, which subsequently evolved to encompass elements with densities greater than 5 g cm^−3^ and atomic weights ranging from 63.5 to 200.6 g mol^−1^. Moreover, it is characteristic for HMIs found in nature to possess a specific gravity over 5 g cm^−3^ [1].

There are many heavy metal ions associated with adverse effects, but the most important include mercury—Hg(II), cadmium—Cd(II), lead—Pb(II), chromium—Cr(III/IV), and arsenic—As(III). The degree of toxicity exhibited by these metals is a function of the exposure route, its duration, and the total absorbed dosage. Entry into the human body occurs through diverse channels, notably via the food chain through plants and fish. Moreover, heavy metals solubilized in water demonstrate a heightened propensity for plant uptake. This process facilitates the bioaccumulation of these metals within plant root systems, followed by their translocation to other consumable plant tissues, including stems, foliage, and fruits. Therefore, human consumption of contaminated fish and plant matter leads to the direct assimilation and subsequent accumulation of heavy metals, ultimately instigating severe adverse health outcomes [3].

Mercury exposure correlates with neurological deficits in infants and cardiovascular impacts in adults. In China, this exposure is implicated in over 10,000 fatal myocardial infarctions each year. Furthermore, in the USA, 75,000 infants are at increased risk of neurological and learning impairments due to mercury exposure. Significant historical incidents of Hg toxicity occurred in Minamata, Japan, and Iraq, where the ingestion of mercury-contaminated fish, shellfish, and grain by local populations resulted in approximately 6000 cases of toxicity in Iraq and 1043 fatalities in Japan [4]. Cadmium is linked to a range of severe health problems. These include emphysema, testicular atrophy, hypertension, renal damage, and skeletal malformations in foetuses. Furthermore, the International Agency for Research on Cancer (IARC) has definitively classified cadmium as a carcinogen, particularly implicated in kidney cancer, based on robust evidence from both human and animal studies [5]. Lead serves no biological purpose in the human body and is harmful even at very small concentrations. Once it enters the body, it can disrupt the metabolism of calcium and other essential nutrients by competing for binding sites. In adults, lead poisoning manifests with various symptoms including headache, increased intracranial pressure, arthralgia, abdominal pain, nephropathy, and nervous system dysfunction. Additionally, lead poisoning has been linked to delayed intellectual development in children [5]. Chromium exists in a wide range of oxidation states, from −2 to +6. In the natural environment, however, it is primarily found as trivalent chromium—Cr(III) and hexavalent chromium—Cr(VI). While chromium in its zero-oxidation state—Cr(0) is biologically inactive and is not naturally present in the Earth’s crust, both Cr(III) and Cr(VI) are commonly introduced into the environment through industrial activities. Cr(III) in small dosages is an essential trace element vital for proper biological function, while Cr(VI) has highly carcinogenic, genotoxic, and mutagenic properties [6,7,8]. Arsenic is a compound whose inorganic forms are considerably more toxic than their organic counterparts, with the most hazardous being As(III). This is due to its strong reactivity with essential enzymes within the human body. Prolonged exposure to As(III) can lead to severe health issues, including skin lesions, keratosis, bladder cancer, and lung cancer [9]. HMIs demonstrably exert significant detrimental effects on both living organisms and environmental integrity. Consequently, continuous environmental monitoring employing highly sensitive analytical methods is imperative.

**Figure 1:**
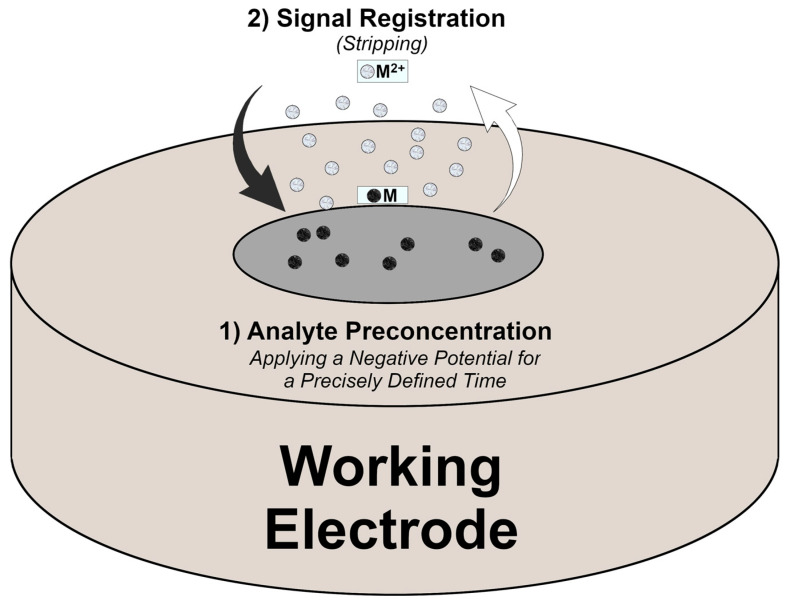
Scheme of HMI measurement using ASV [16,19].

Among many instrumental techniques, such as chromatographic [10,11,12] or spectroscopic [13,14,15] methods, electroanalytical methods, such as voltammetry, have many advantages, including low equipment costs, short analysis time, low measurement expense, and the possibility of multi-element analysis at the trace concentration level. This is possible thanks to the use of stripping voltammetry (SV) technique. SV is characterised by high sensitivity thanks to an additional step of analyte accumulation (usually with applying the accumulation potential) before the signal recording stage (involving a potential scan), when the deposited ions are stripped back into the solution (Figure 1) [16]. Before the next measurement, an additional electrochemical cleaning step is often performed by applying a positive potential to the electrode for a certain period. Various forms of SV can be distinguished based on the characteristics and direction of the preconcentration and measurement stages, such as (a) anodic stripping voltammetry (ASV), (b) cathodic stripping voltammetry (CSV), and (c) adsorptive stripping voltammetry (AdSV). The first utilizes cathodic preconcentration followed by a scan towards more positive potentials. In CSV, the analyte is accumulated in the form of insoluble salts and subsequently reduced to obtain signals. In AdSV, the analyte is adsorbed onto the electrode surface and reduced due to a potential scan towards more negative potentials. The stripping stage can be performed using various voltammetric waveforms, of which the most popular are square wave (SW) and differential pulse (DP). SW is the most rapid technique, but DP is more sensitive [17]. The selection of the appropriate technique is also predefined by the rate of reaction kinetics and iteration between the analyte and the electrode surface. In the literature, the most used techniques in direct analysis of HMIs are those based on the conjunction of SV and DP or SW: square wave anodic stripping voltammetry (SWASV) and differential pulse anodic stripping voltammetry (DPASV) [18].

Although the development of voltammetry has been going on since the first half of the twentieth century, this technique still has a lot to offer [20]. The most important element of each voltammetric procedure is the working electrode (WE), the surface of which interacts directly with the analyte. For this reason, the current development path of voltammetry is towards matching the surface properties of the WE to the properties of the analyte by various modifications [21,22,23,24].

Since R.P. Feynman’s (Nobel Prize winner) breakthrough in 1959, nanotechnology has gained popularity and retains it to this day [18,25]. Recently, nanomaterials such as magnetite (Fe_3_O_4_) magnetic nanoparticles (MNPs) are gaining popularity due to their beneficial properties. They are characterized by biocompatibility, low toxicity, very good conductivity, chemical stability, supermagnetism, and very good adsorption and catalytic properties towards HMIs [5,21,26,27,28,29,30]. Therefore, Fe_3_O_4_ MNPs seem to be an excellent electrode modifier for voltammetric trace analysis of HMIs.

This article presents a condensed review of functionalized Fe_3_O_4_ magnetic nanoparticles from the methods of their synthesis and modification of different types of electrode materials to their application in the voltammetric trace analysis of HMIs. This paper describes the effect of electrode surface modification strategies on the stability of MNPs and the homogeneity of their distribution on the carbonaceous carrier surface. The voltammetric procedures for the determination of HMIs using different electrode materials modified with Fe_3_O_4_ magnetic nanoparticles are compared and discussed. It should be emphasized that the work is an extension of the already published review [18], taking into account the wider scope of works, including papers published after 2021 and various electrode materials (not only the glassy carbon electrode (GCE)). Moreover, this review supplements that work with current trends observed in Fe_3_O_4_-based sensor preparation, the most common Fe_3_O_4_ MNPs synthesis protocols and their comparison, and modification strategies, as well as a very broad benchmark of analytical procedure performance, with particular emphasis on the type of electrolyte, measurement technique, and application of the procedures for single and multi-element analysis published in the years 2012–2025.

## 2. The Properties, Synthesis, and Modification Strategies of Fe_3_O_4_ MNPs

### 2.1. The Insight into Magnetite Properties

Ferro- and ferrimagnetic materials include metals such as iron (Fe), cobalt (Co), and nickel (Ni), as well as oxides and alloys of these metals. However, of all available magnetic nanomaterials, iron oxides, which can occur in 16 varieties, including hematite (α-Fe_2_O_3_), magnetite (Fe_3_O_4_), and maghemite (γ-Fe_2_O_3_), are the most popular. Of these three, magnetite (also known as black iron oxide or lodestone) is the strongest magnetic ion, while hematite is the weakest [30,31,32].

Magnetite is a black solid with a density of 5.18 g cm^−3^. It is characterized by average hardness (5 on the Mohs scale) as well as high melting (1583–1597 °C) and boiling (2623 °C) points. Fe_3_O_4_ exhibits ferrimagnetism at room temperature and loses magnetic properties after exceeding a temperature of 850 °C (Curie temperature) [18]. The properties of nanomaterials, such as iron nanoparticles, greatly depend on size, phase, and morphology, which can be controlled through synthesis methods [31]. Magnetite is characterized by biocompatibility, low toxicity, excellent conductivity, chemical stability, and very good adsorption and catalytic properties towards HMIs [5,21,26,27,28,29]. Fe_3_O_4_ magnetic nanoparticles contain trivalent and bivalent Fe in their structure, placed at interstitial locations. The iron atoms are located in a cubic inverse ridge configuration. The oxide ions are arranged in the octahedral position in a face-centred cubic (FCC) lattice. Very good adsorption of HMIs and high electron transfer properties of magnetite are provided thanks to the position of 16 trivalent Fe^3+^ in tetrahedral and octahedral positions as well as the distribution of 8 divalent Fe^2+^ in the octahedral position. The magnetite molecular formula is written as follows: (Fe^2+^)(Fe^3+^)_2_O_4_ [26,31,33,34].

Fe_3_O_4_ nanoparticles are characterized by a high surface area to total mass ratio, which makes them sensitive to the reduction-oxidation processes, pH changes, or the effects of aggressive reagents. Unfortunately, bare Fe_3_O_4_ is unstable in an acidic environment [30]—the conditions in which voltammetric determinations of HMIs are most often performed. Moreover, MNPs are very susceptible to the oxidation process, especially when they have dimensions of up to 100 nm, which leads to the transformation of magnetite into maghemite nanoparticles as shown in the reaction below [30,34]:

Fe_3_O_4_ + 2H^+^ → γ-Fe_2_O_3_ + Fe^2+^ + H_2_O (1)

In the presence of H_2_O and O_2_, transformation of magnetite to iron hydroxide occurs, which extensively changes the magnetic and chemical properties of MNPs [30,34]:

Fe_3_O_4_ + 0.25O_2_ + 4.5H_2_O → Fe(OH)_3_ (2)

Bare Fe_3_O_4_ nanoparticles tend to spontaneously form of nanoparticle aggregates due to their high surface energy, resulting in a significant decrease in catalytic sites [5,27,35]. Moreover, the loss of magnetism caused by the oxidation of iron ions Fe^2+^ to Fe^3+^ is observed. Additionally, an insufficient amount of surface functional groups capable of interacting with the analyte can cause problems of poor sensitivity [30,36]. Therefore, modification of magnetic nanoparticles is crucial to eliminate these problems, which is described in Section 2.3.

### 2.2. The Synthesis of Fe_3_O_4_ Magnetic Nanoparticles

Several synthesis methods can be used to produce MNPs, including co-precipitation, sonochemical, ball milling, laser ablation, sol-gel, and hydrothermal methods [30,31]. The literature review of many Fe_3_O_4_-based voltammetric techniques for the determination of HMIs indicated that among these methods, the most popular, least complicated, most effective, and most widely used are different variations of the co-precipitation [5,7,23,37,38,39,40,41,42] and hydrothermal/solvothermal methods [24,26,33,36,43,44,45].

The co-precipitation method generally involves the addition of base to provide an alkaline environment, which produces Fe_3_O_4_ MNPs in a salt solution (Fe^2+^/Fe^3+^) [46]. There are two strategies in synthesizing MNPs: (1) using different oxidizing agents that simultaneously oxidize Fe(OH)_2_, and (2) converting initially formed MNPs to aging Fe(OH)_2_ and mixtures of Fe_3_O_4_ in the changing medium. In addition to the type of salt used, the size of the iron oxide depends on various parameters, such as reaction temperature, pH, ionic strength, stirring speed, and solution addition. The pH must be kept in the range of 8–14, and it is important to adjust the ionic strength and medium pH to control the particle size and maintain the Fe^3+^/Fe^2+^ ratio at 2/1. This synthesis approach is used to produce MNPs for all demulsification applications involving emulsions [31,47].

The hydrothermal method presents several key benefits, e.g., reducing particles agglomeration and creating uniform, homogeneous crystals. Notably, it facilitates the enhancement of nanoproperties in high-temperature environments and can diminish nano-activity under high-pressure conditions. The method is also valued for promoting environmental protection and ensuring product purity. Nevertheless, the solution’s stoichiometry control could be challenging. The operational process is straightforward, relying on cost-effective materials with minimal environmental impact, and involves a reactor designed for controlled high-temperature and high-pressure external conditions. Furthermore, it is particularly effective for producing substances with low solubility or insolubility. The synthesis pathway generally comprises recrystallization, followed by separation and heat treatment to yield nanoparticles [31].

**Figure 2:**
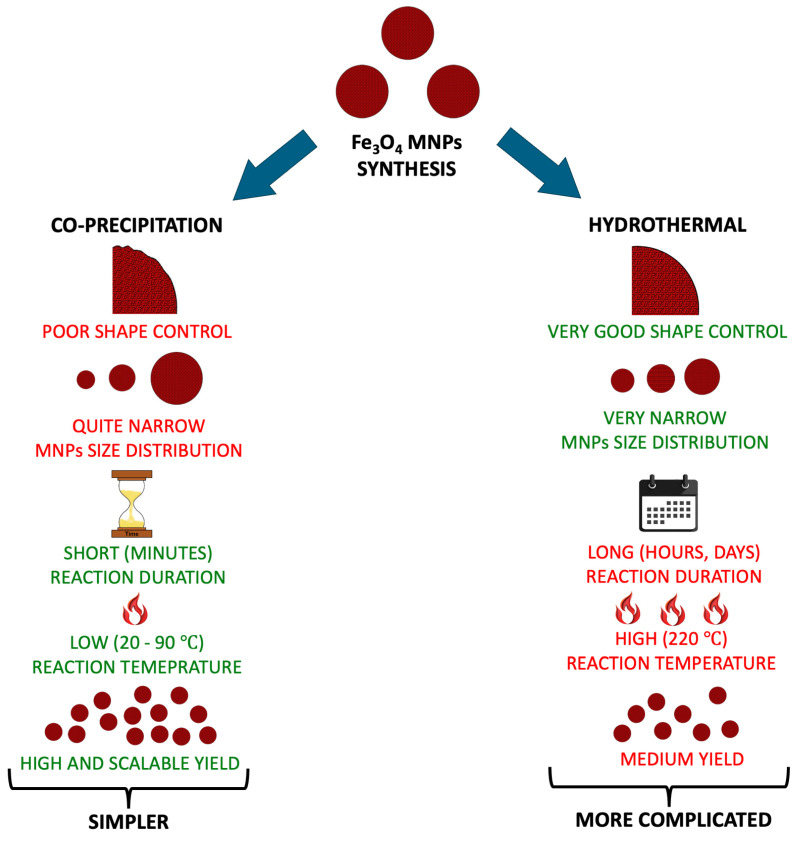
Comparison of the features of the co-precipitation and hydrothermal methods [48].

Figure 2 summarizes the most popular methods for the synthesis of Fe_3_O_4_ nanoparticles in voltammetric application (HMIs analysis). The co-precipitation is more straightforward than the hydrothermal method. Moreover, this method is conducted at a lower temperature in an ambient condition, the reaction time takes only a few minutes, and it allows for obtaining MNPs with very high, scalable yield. Although the hydrothermal method fares worse in these fields, its advantage over the co-precipitation method is a better control over the synthesis parameters, such as magnetic nanoparticles’ size distribution and shape control [48].

### 2.3. The MNPs Modification Strategies

Several surface modification strategies of Fe_3_O_4_ magnetic nanoparticles can be distinguished, such as: (1) encapsulation—surrounding iron oxide nanoparticles resulting in the formation of core/shell nanostructures, in which both layers differ significantly in chemical composition, and the final hybrid nanomaterials exhibit properties that are collectively given to them through both layers; (2) matrix-dispersed structures—magnetic iron oxide nanoparticles can be dispersed in the matrix (coating substance); (3) Janus structures—two-element structures in which one part of the nanoparticle is Fe_3_O_4_, while the second one is a functionalized material (e.g., Pt-Fe_3_O_4_); and (4) shell/core/shell structures—magnetic iron oxide nanoparticles are located between two layers of functionalizing materials [30].

The most effective method of magnetic nanoparticles surface modification is the encapsulation process, which increases the stability of nanoparticles, reduces their susceptibility to aggregation, and ensures appropriate dispersity. Thanks to the process of Fe_3_O_4_ MNP-coating, the decomposition and loss of physicochemical properties are prevented [48,49]. Coating layers can be divided into inorganic and organic materials. Organic modifiers such as surfactants, polymers, small molecules, and biomolecules tend to protect Fe_3_O_4_ MNPs from aggregation and adjust their surface charge and hydrophobicity, thereby enhancing its stability and biocompatibility. Obviously, the surfaces of these modifiers contain abundant functional groups such as amines, thiols, hydroxyl, and carboxylic acid, etc., which combine with the (–OH) of Fe_3_O_4_ MNPs to form monodisperse composite nanoparticles through electrostatic adsorption or covalent chelation, which also affect the adsorption properties of HMIs [5,35,50]. Modification of Fe_3_O_4_ surface with citrate (citrate@Fe_3_O_4_) proposed by Qureashi et al. [23] increased the hydrophobicity of unmodified Fe_3_O_4_ MNPs and incorporated abundant hydroxyl and carboxyl groups onto the magnetite surface. Consequently, it enhanced the adsorption capacity of Pb^2+^ ions. In contrast, inorganic modifiers such as silica, graphene, metals, and metal oxides tend to protect Fe_3_O_4_ from corrosion by other reagents, thus strengthening its stability and catalytic activity [30,35,51]. For example, silica (SiO_2_) is an ideal surface modifier for Fe_3_O_4_ MNPs, since its surface groups are easily activated, and Si–O–Si bonds can also combine with Fe_3_O_4_ MNPs to form stable Fe–O–Si chemical bonds [48]. The resulting core/shell structures constitute a starting point for further modifications, the purpose of which is to give magnetic nanoparticles appropriate functions and properties.

**Figure 3:**
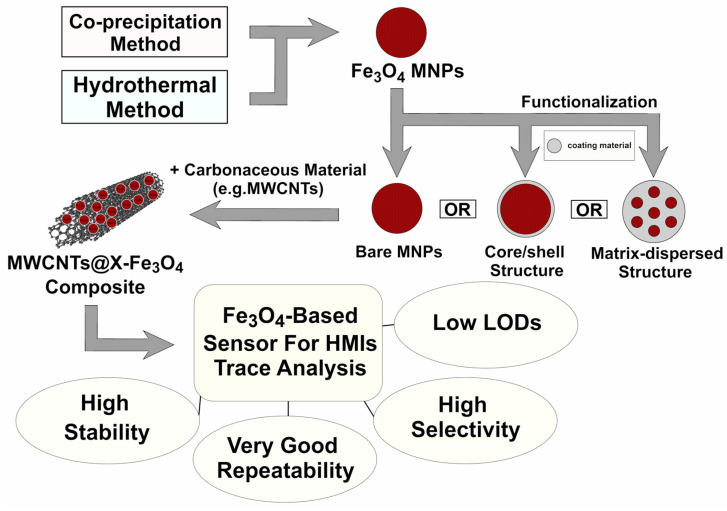
Schematic representation of the most common steps in preparing the sensors based on Fe_3_O_4_ nanoparticles [24,27,41,52,53].

Another key solution is to use a conductive nanomaterial as a support. Carbon materials, especially multi-walled carbon nanotubes (MWCNTs), are promising candidates as supporting materials because of their large specific surface area, electrical conductivity, and high chemical stability. The introduction of MWCNTs can reduce the Fe_3_O_4_ aggregation, expose more active sites for detection, enhance the electron transfer rate, increase the catalytic activity by synergy, and thus improve the HMIs detection sensitivity [26,27,52]. Figure 3 shows a schematic representation of the most common steps in preparing the sensors based on Fe_3_O_4_ nanoparticles [24,27,41,52,53].

## 3. Voltammetric Determination of HMIs via Fe_3_O_4_-Based Sensors

### 3.1. The Type of Support and Procedure for Working Electrode Preparation

Most of the published studies on voltammetric HMIs determination procedures using sensors based on Fe_3_O_4_ nanoparticles utilize carbonaceous material as a base for modification. The glassy carbon electrode (GCE) and its magnetic variant (magnetic glassy carbon electrode—mGCE), the magnetic carbon paste electrode (mCPE), and different variations of screen-printed electrodes (SPEs) are usually used (see Table 1, Table 2 and Table 3). Among these electrodes, the GCE/mGCE are the most popular. However, the conventional electrodes such as GCE/mGCE and CPE/mCPE are not suitable for small-volume analysis due to their relatively large size. This is where flat and miniaturized SPEs come into play, mainly in flow systems or portable analysers.

Glassy carbon material is obtained by pyrolysis in an inert atmosphere (nitrogen or argon, etc.) of carbonaceous resins. This process is conducted with different variations of precursors, heating, and atmosphere conditions to fine-tune material properties. Thanks to this treatment, a dense, non-graphitic structure with excellent durability and stability is obtained [54]. The mGCE is a GCE with an additional magnet attached, which could reduce the loss of magnetic material deposited on its surface (such as Fe_3_O_4_) due to the surface modification and improve the stability of prepared sensors [53,55]. Some studies utilize homemade mGCE electrodes, which are made by mixing paraffin oil and graphite powder. The prepared paste is inserted into a Teflon tube, and the copper contact is introduced into the paste. Next, a coin-shaped magnet and a glassy carbon electrode disc, respectively, are inserted into the tube. Finally, the glassy carbon disc is sealed and polished [5,53,56]. Thanks to wide availability and high quality, commercially offered GCEs and mGCEs are usually used in scientific research, most commonly with a diameter of 3 mm. Glassy carbon-based electrodes should always be properly polished before application of the modifying material. There are many ways to do this properly, but commonly a GCE is polished to a mirror-like surface with wet 0.3 μm and 0.05 μm Al_2_O_3_ powder using a suede cloth and washed with ethanol and deionized water for 5 min each [33]. In some works, additional polishing stages utilizing silicon carbide paper, aluminium oxide of different grammage, different sonication times, and various types of solutions used for ultrasonic cleaning (e.g., HNO_3_ (1:1, v/v)) are used. After the polishing process, the electrode is dried using air or abundant gas (e.g., N_2_) [5,9,26,38,45]. The ink with modifying agents is usually prepared in water, DMF, acetic acid, or alcohol (e.g., ethanol or isopropyl alcohol) and homogenized in an ultrasonic bath to form a homogeneously dispersed solution. The studied material is usually drop-casted onto the polished GCE/mGCE surface and dried using air, an infrared lamp, or a laboratory dryer [23,24,26,38,44,52,53,57].

The carbon paste electrode is another widely used type of electrode in voltammetry. The preparation process includes mixing paraffin oil and graphite powder in a mortar. Afterward, the paste is inserted into a plastic tube and connected with a copper contact. The surface is renewed by the replacement of the carbon paste as well as polishing on weighing paper. The magnetic variant is prepared by placing a magnet behind the working part of the sensor. This electrode is subsequently modified by coating of mCPE surface with the ink consisting of, for example, a water-alcohol suspension of modifiers [37,40].

Other sensors characterized by high portability, low cost, and excellent reproducibility are screen-printed electrodes (SPEs). They are usually equipped with a system of three electrodes, such as a working electrode (WE), a pseudo-reference electrode (pRF), and an auxiliary electrode (AUX). Currently, they are widely, commercially available, but they can be also be self-prepared quite simply. The SPE manufacturing process begins with mesh design. This step is crucial for the size and geometry of the electrode. Selection of conductive ink and substrate materials is determined for the designed application, but carbon-based material is the most popular for WE and AUX preparation. The pseudo reference electrode is usually made from silver or silver chloride inks. To prepare durable, uniform, and stable electrodes, the layer-by-layer technique is used for their preparation, and subsequently, the sensors are solidified using hot air and IR radiation. Finally, electrical circuits are insulated, and the final sensors can be modified using, for example, a drop casting method [53,58,59]. The magnetic field from an external magnet also can be used to stabilize the drop-casted ink on the SPE surface [60]. The deposition of metallic film (e.g., bismuth film) onto the final sensor’s surface is also a practiced strategy of modification [61].

### 3.2. The Application of Fe_3_O_4_ MNPs-Based Sensors in Trace Voltammetric HMIs Analysis

Many voltammetric procedures for determination of HMIs such as Ag(I), Cd(II), Pb(II), Hg(II), Cu(II), Zn(II), As(III), and Cr(III/IV), using sensors based on Fe_3_O_4_ magnetic nanoparticles have been presented in the literature. Most of these are characterized by a wide range of linearity of calibration curves and low detection limits both in the analysis of single ions (Table 1), simultaneous determination of two HMIs (Table 2), and in multi-element analysis (Table 3). The analytical procedures were used in the analysis of liquid matrices (river water, lake water, dam water, pool water, groundwater, aqueduct water, well water, tap water, mineral water, spring water, wastewater, rain water, laboratory water, industrial effluents, milk, orange juice, and red wine samples [5,9,24,26,27,33,36,37,38,40,41,42,43,44,45,52,53,57,60,62,63,64,65,66,67,68,69,70,71]) as well as solid samples (rice, soybean, soil, and fish samples [36,60,61]).

In most cases, the optimized procedures utilize acidic electrolytes of which the most popular is the 0.1 mol L^−1^ acetate buffer with a pH of approximately 4.0–5.0, what was well summarized in the work of Kulpa-Koterwa et al. [18]. The authors of that review suggest that the increase of electrochemical signals of measured ions observed in the acetate buffer solution is caused not only by the appropriate pH but also because the acetate buffer enables a binding reaction as a result of intermolecular ion binding on the surface of the Fe_3_O_4_-modified electrode. Only selected procedures for single elemental analysis (Table 1) utilize an electrolyte with neutral or neutral-close pH such as KCL (Ag^+^) [65], NaNO_3_ (Pb^2+^) [23], and PBS pH = 7.5 (Hg^2+^) [60]. Measurements in non-direct analysis can also be performed using [Fe(CN)_6_]^3/4−^ and PBQ/H_2_Q + NaClO_4_ of pH 3.5 probes [7]. To sum up, an acidic environment is the most suitable condition for HMIs determination. Due to the metal-ligand speciation changes, an increase in pH, especially above the neutral one, leads to the creation of hardly soluble species, e.g., hydroxides. As a result, a reduction of the stripping peak is observed as fewer metal ions are deposited in the metallic state on the electrode surface. Nevertheless, extremely low pH can alter the physicochemical properties of Fe_3_O_4_ material and diminish the obtained signals [18,23,72].

The selected examples of analytical approaches for the determination of individual ionic species (Table 1), simultaneous analysis of two HMIs (Table 2), and comprehensive multi-elemental determinations (Table 3) are further discussed. He et al. [24] presented a procedure for determination of Pb(II) ion using a glassy carbon electrode modified with graphene/Fe_3_O_4_ nanosheets functionalized with garlic extract (Fe_3_O_4_/GN/GE/GCE) having wide linear ranges: 1.0 × 10^−12^–5.0 × 10^−10^ mol L^−1^ and 5.0 × 10^−10^–1.0 × 10^−6^ mol L^−1^. Moreover, their procedure is characterized by an extremely low LOD = 1.23 × 10^−14^ mol L^−1^ (the lowest LOD among all the HMIs determination procedures discussed in this review article) as well as by high stability and reproducibility. To evaluate the reproducibility of a sensor, the authors tested the consistency of its response. They used eight different electrodes and measured their signal in the presence of 1000 nM of Pb^2+^. The results showed a low variation of just 3.61%, confirming the method’s excellent reproducibility. The stability of the sensor was also tested over 10 weeks. Between the uses, the sensor was kept in a refrigerator at 4 °C. Measurements were taken weekly, and after the 10-week period, the signal response remained largely unchanged, with a relative standard deviation (RSD) of 6.28%. The sensor retained 86.14% of its original signal for detecting 1000 nM of Pb^2+^. Moreover, in this work the authors emphasize the important role of well-conductive, porous graphene nanosheets (GNs) with a high active surface area as a support for Fe_3_O_4_ magnetic nanoparticles, preventing their aggregation and maintaining their activity. The modification with garlic extract strongly anchored Fe_3_O_4_ MNPs on the GNs, which can form strong complex with lead ions, thus providing more active sites for Pb(II) accumulation. The authors verified procedure’s specificity in the presence of Ag(I), Co(II), Cu(II), Zn(II), Mn(II), Ni(II), Hg(II), Cd(II), and Fe(III) ions, and proved to be suitable for the analysis of real samples. The developed procedure was used for the analysis of Pb(II) ions in tap water, rain water, river water, and industrial effluents [24]. Baghayeri et al. [41] developed a procedure for individual and simultaneous analysis of Cd(II) and Pb(II) ions using a glassy carbon electrode modified with poly(amidoamine) dendrimer functionalized magnetic graphene oxide (GO-Fe_3_O_4_-PAMAM/GCE). The procedure is distinguished by a wide single linear range of the calibration curve for Cd(II)—1.78 × 10^−9^–1.25 × 10^−6^ mol L^−1^ and Pb(II)—1.93 × 10^−9^–5.79 × 10^−7^ mol L^−1^. Moreover, this method allows to obtain very low LODs to be obtained for both Cd(II) and Pb(II) equal to 6.20 × 10^−10^ mol L^−1^ and 6.30 × 10^−10^ mol L^−1^, respectively. The sensor’s reproducibility was confirmed by testing eight different electrodes with a solution containing 20 mg L^−1^ of each individual ion, yielding low relative standard deviation (RSD) values of 3.5% for Cd(II) and 4.0% for Pb(II). Additionally, the sensor’s repeatability in the presence of mixed 20 mg L^−1^ Cd(II) and Pb(II) solution was excellent, resulting in very low RSD values of 1.7% for Cd(II) and 2.1% for Pb(II) from the stripping currents. The sensor also demonstrated long-term stability, maintaining its performance after being stored dry at room temperature for three weeks. During this period, it retained 96.3% of its initial response for Cd(II) and 95.7% for Pb(II), confirming its reliability over time. The developed sensors utilize the advantages of the combination of carbonaceous material and magnetite and modification with multiple functional group-rich PAMAM dendrimers. The procedure is quite resistant to the presence of interfering species such as Tl(I), Ni(II), Cu(II), Zn(II), Hg(II), Co(II), Ca(II), Mg(II), Cd(III), In(III), Fe(III), and Mn(III), and has been successfully used to analyse Cd(II) and Pb(II) in lake and river water [41].

The paper by Wu et al. [27] presented the procedure for simultaneous analysis of Cd(II), Pb(II), Cu(II), and Hg(II) using a glassy carbon electrode modified with Nafion, Fe_3_O_4_ nanoparticles, and multi-walled carbon nanotubes (F-MWCNTs/Fe_3_O_4_/Nafion/GCE). The procedure is distinguished by very wide linear range equal to 5.00 × 10^−10^–3.00 × 10^−8^ mol L^−1^ for Cd(II), Pb(II), and Cu(II), as well as 5.00 × 10^−10^–2.00 × 10^−8^ mol L^−1^ for Hg(II). The obtained limits of detection for those ions were equal 5.00 × 10^−11^ mol L^−1^, 8.00 × 10^−11^ mol L^−1^, 2.00 × 10^−11^ mol L^−1^, and 5.00 × 10^−11^ mol L^−1^, respectively. The authors enhanced the electronegativity and conductivity of well-conductive MWCNTs by fluorination, which together with Fe_3_O_4_ MNPs, provided a sufficient number of active sites suitable for multi-element analysis. The developed procedure has decent resistance to the presence of metal ions such as K(I), Ca(II), Co(II), Ba(II), Ni(II), Mn(II), and Zn(II), and can be used for analysis of these ions in river water and soybeans [27]. Our group also worked on the development of a sensor for the simultaneous determination of Cd(II) and Pb(II) ions at trace concentration levels. For this purpose, a glassy carbon electrode modified with Nafion, MWCNTs, polyethylene glycol functionalized magnetite, and bismuth film (GCE/Nafion/MWCNTs@PEG-Fe_3_O_4_/BiF) was used [52]. The developed procedure is distinguished by a very short, 120 s deposition time, linear ranges from 2.0 × 10^−9^– 5.0 × 10^−7^ for both ions, low LODs equal to 4.90 × 10^−10^ mol L^−1^ and 4.60 × 10^−10^ mol L^−1^, and high sensitivities of 85.14 μA/μmol L^−1^ and 74.21 μA/μmol L^−1^ for Cd(II) and Pb(II), respectively. Moreover, in this paper we examined the effect of every individual element of the composite (BiF, Nafion, MWCNTs and PEG-Fe_3_O_4_) on the signal of Cd(II) and Pb(II). The studies revealed that every composite component had positive influence on the signal of both ions in comparison to the GCE/BiF electrode, except for the sensor using GCE/Nafion/PEG-Fe_3_O_4_/BiF without carbon support. In this case, we observed increase of Pb(II) and a decrease of the Cd(II) signal. This effect is caused by higher affinity of Pb(II) ions to the presence of hydroxyl groups on the surface of Fe_3_O_4_ MNP, provided by polyethylene glycol modification, than Cd(II) ions. However, the addition of well-conductive and porous MWCNTs provided an additional number of active sites available for detection for both ions. Physicochemical studies (cyclic voltammetry and electrochemical impedance spectroscopy measurements in the redox system) revealed that PEG-Fe_3_O_4_ addition to the composite composition decreased the charge transfer resistance, increased the charge transfer rate, and slightly increased the active surface area of the tested sensor. The addition of MWCNTs increasingly improved those parameters as well. Thanks to the scanning electron microscopy images (SEM), we confirmed that combining magnetite nanoparticles with the carbon material improved the homogeneity of the nanoparticle distribution on the sensor surface [52]. It is worth noting that the method of electrode drying after dropping the ink onto the surface of the GCE can affect the sensor’s stability over time. In the case of this procedure [52], drying the GCE/Nafion/MWCNTs@PEG-Fe_3_O_4_ composite in a laboratory dryer before electrochemical deposition of the bismuth film allowed obtaining electrodes with stable, repeatable signals up to three weeks after sensor preparation [52].

Simultaneous determination of many elements in voltammetry is a very difficult task, burdened with many problems including the competitive deposition of the determined ions, the creation of intermetallic compounds, an insufficient number of active sites, and the risk of sensor clogging [27,68]. For example, Zhang and Guo [68] compared the analytical performance of their magnetic carbon electrode coated with TEOS modified Fe_3_O_4_ magnetic nanoparticles (Fe_3_O_4_@SiO_2_/mGCE) towards individual and simultaneous analysis of Cd(II), Pb(II), Cu(II), and Hg(II) ions. Their DPSV results indicate deterioration of the analytical parameters for the simultaneous analysis in comparison to the measurements performed individually. However, the authors obtained low LODs equal to 5.61 × 10^−8^, 1.65 × 10^−8^, 7.94 × 10^−8^, and 5.67 × 10^−8^ mol L^−1^ and wide linear ranges of the calibration curves equal to 1.00 × 10^−7^–1.00 × 10^−4^, 1.00 × 10^−7^–8.00 × 10^−5^, 1.00 × 10^−7^–8.00 × 10^−5^, and 1.00 × 10^−7^–1.00 × 10^−4^ µM for the simultaneous analysis of Cd(II), Pb(II), Cu(II), and Hg(II), respectively. Moreover, the selectivity studies showed the procedure to have decent resistance to the presence of 5× excess of foreign ions such as Mg(II), Fe(III), Co(II), Ni(II), Mn(II), Zn(II), Ca(II), and Al(III), with only a 2.33–6.66% signal decrease. Finally, the obtained sensor showed good repeatability (0.82–2.13% RSD) [68].

A double electrode system, with each electrode dedicated to the accumulation and stripping of specific metal ions, could be a method to boost the analytical capabilities in multi-element analysis. The procedure proposed by Zhao et al. [69] utilizes this system. A screen-printed electrode modified with Nafion and (BiO)_2_CO_3_-reduced graphene oxide ((BiO)_2_CO_3_-rGO-Nafion/SPE) was used for simultaneous determination of Cd(II) and Pb(II), and a screen-printed electrode modified with Au and Fe_3_O_4_ magnetic nanoparticles and ionic liquid (Fe_3_O_4_-Au-IL/SPE) served as a sensor for As(III) determination. The measurements were performed using the flow analysis regime. The deposition time and potential were optimized individually for both sensors. The authors studied surface morphologies of bare SPE, (BiO)_2_CO_3_-rGO-Nafion/SPE and Fe_3_O_4_-Au-IL/SPE using SEM. After modification of bare SPE, the electrode morphology changed drastically, as evident by the abundance of bismuth or gold nanoparticles on the electrode. In addition, the AuNPs are covered with Fe_3_O_4_ NPs, which can be expected to obtain a high catalytic performance for As(III) [70].

**Figure 4:**
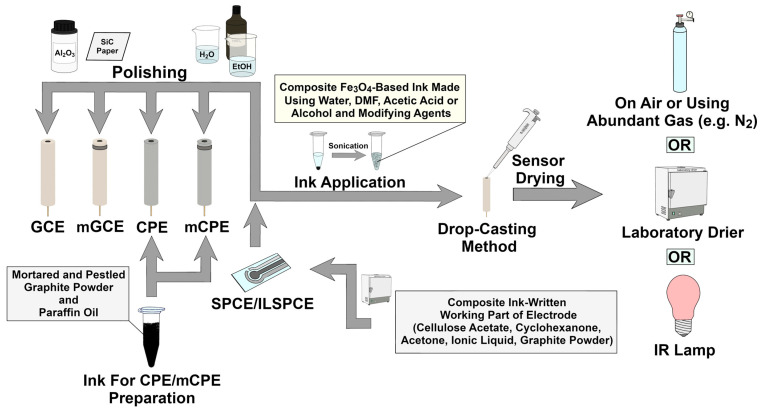
The most effective strategy for creating a sensitive and selective sensor based on Fe_3_O_4_ MNPs for trace analysis of HMIs [24,27,41,52].

As mentioned before, the procedures presented in this work are characterized by very good analytical parameters (very low LODs, wide linear ranges of calibration curves) and were used to selective analysis of HMIs in various samples. Moreover, described procedures are characterised by sensors stable for a long time (even ten weeks, with 13.86% signal loss compared to the initial signal) and very good repeatability (RSD < 6.3%) [24,26,27,41,68]. Figure 4 sums up the most effective strategy for creating a sensitive and selective sensors based on Fe_3_O_4_ MNPs for trace analysis of HMIs, which is also characterized by high repeatability and stability. It should be noted that the lowest LODs were generally obtained for the above-mentioned procedures and sensors using carbonaceous material as a support for Fe_3_O_4_ nanoparticles. This indicates the important role of the functionalized-Fe_3_O_4_-carbonaceous support combination in the design of future state-of-the-art sensors (which would achieve the lowest LODs, the widest linear ranges, and the highest selectivity) for trace determination of HMIs.

**Table 1:** Selected examples of voltammetric procedures for the single HMI determination using sensors based on Fe_3_O_4_ magnetic nanoparticles.

Sensor	Technique	Base Electrolyte Composition	Analytical Parameters [mol L^−1^]	Determined Ion [mol L^−1^]	Application	Ref.
Determination of Ag(I):			Ag(I)			
Fe_3_O_4_@SiO_2_@IIP/CPE	DPASV	0.1 mol L^−1^ HCl	LOD * Linear range *	1.39 × 10^−10^ 4.64 × 10^−10^–1.39 × 10^−6^	dam water, aqueduct water, and well water	[37]
Fe_3_O_4_@Au/mGCE	DPAV	0.1 mol L^−1^ KCl	LOD Linear range	5.9 × 10^−10^ 1.17 × 10^−7^–1.77 × 10^−5^	tap water, lake water, and synthesized water	[65]
Determination of Cd(II):			Cd(II)			
BiF/Fe_3_O_4_/ILSPE	DPASV	0.2 mol L^−1^ PBS pH = 5.0	LOD * Linear range *	4.45 × 10^−10^ 4.45× 10^−9^–3.56 × 10^−7^	soil samples	[61]
Fe_3_O_4_-PEI-Au-SPCE-Apt	DPV	-	LOD Linear range	1.00 × 10^−11^ 4.00× 10^−11^–2.50× 10^−8^	drinking water, dam water, river water, and wastewater	[62]
Determination of Pb(II):			Pb(II)			
Fe_3_O_4_@Citrate/GCE	DPASV CV	0.1 mol L^−1^ NaNO_3_	LOD Linear range	3.0× 10^−7^ 5.0 × 10^−7^–1.5× 10^−5^	-	[23]
Fe_3_O_4_@PDA@MnO_2_/mGCE	DPV	1.0 mol L^−1^ HCl	LOD * Linear range *	1.4 × 10^−10^ 4.8 × 10^−10^–7.2× 10^−7^	lake water samples	[43]
Fe_3_O_4_/GN/GE/GCE	SWASV	0.1 mol L^−1^ CH_3_COOH/CH_3_COONa pH = 5.5	LOD Linear range	1.23 × 10^−14^ 1.0 × 10^−12^–5× 10^−10^ 5.0 × 10^−10^–1.0× 10^−6^	tap water, rain water, river water, and industrial effluents	[24]
Determination of Cu(II):			Cu(II)			
Fe_3_O_4_/SiO_2_/CS/Nafion/GCE	DPASV	0.1 mol L^−1^ PBS pH = 4.0	LOD Linear range	5.0 × 10^−9^ 1.0 × 10^−8^–2.0× 10^−5^	river and tap water	[44]
Determination of Hg(II):			Hg(II)			
Fe_3_O_4_@Au/CA/T-COOH/SPCE	DPASV	0.01 mol L^−1^ PBS pH = 7.5	LOD * Linear range *	2.41 × 10^−9^ 4.83 × 10^−9^–9.65× 10^−7^	environmental water, wastewater, certified reference material, and organic samples, such as fish samples	[60]
mCPE/HNTs-Fe_3_O_4_–MnO_2_	DPV	0.1 mol L^−1^ HCl	LOD * Linear range *	1.0 × 10^−9^ 2.49 × 10^−9^–7.48 × 10^−7^	water samples	[66]
Determination of As(III):			As(III)			
mGCE/GO-g-Fe_3_O_4_-PAMA-COOH/AuNPs	SWASV	0.1 mol L^−1^ CH_3_COOH/CH_3_COONa pH = 5.0	LOD * Linear range *	6.27 × 10^−9^ 1.33 × 10^−8^–1.67× 10^−6^	tap water, mineral water, and groundwater	[53]
Au NPs/Fe_3_O_4_/GCE	SWASV	0.1 mol L^−1^ PBS pH = 5.0	LOD * Linear range *	1.29 × 10^−11^ 1.33 × 10^−10^–1.33× 10^−8^	tap water, spring water, and lake water	[9]
Determination of Cr(III)/Cr(IV)				Cr(III) or Cr(IV)		
mCPE/MNPZ/Cr(III)	CV	0.5 mmol L^−1^ [Fe(CN)_6_]^3-/4-^ + 0.1 mol L^−1^ NaClO_4_ + 0.1 mol L^−1^ PBS of pH = 3.5 probe	LOD Linear range	8.05 × 10^−10^ 1.00 × 10^−9^–1.00× 10^−3^	-	[7]
	EIS		LOD Linear range	6.90 × 10^−11^ 1.00 × 10^−9^–1.00× 10^−6^		
mCPE/MNPZ/Cr(VI)	CV	5.0 mmol L^−1^ PBQ/H_2_QH_2_O + 0.1 mol L^−1^ NaClO_4_ + 0.1 mol L^−1^ PBS of pH 4.0 probe	LOD Linear range	5.30 × 10^−10^ 5.00 × 10^−9^–1.00× 10^−4^		
	EIS		LOD Linear range	4.46 × 10^−10^ 5.00 × 10^−9^–5.00× 10^−5^		

**Techniques:** DPASV—differential-pulse anodic stripping voltammetry; DPV—differential-pulse voltammetry; SWASV—square-wave anodic stripping voltammetry; CV—cyclic voltammetry. **Sensors:** Fe_3_O_4_@SiO_2_@IIP/CPE—carbon paste electrode modified with silver-imprinted polymer based on silica-coated Fe_3_O_4_ magnetic particles; Fe_3_O_4_@Au/mGCE—magnetic glassy carbon electrode modified with Fe_3_O_4_@Au magnetic particles; BiF/Fe_3_O_4_/ILSPE—screen-printed electrode decorated with ionic liquid (IL), magnetite nanoparticles (Fe_3_O_4_), and coated with a bismuth film (BiF); Fe_3_O_4_-PEI-Au-SPCE-Apt—screen-printed carbon electrode modified with gold nanoparticles decorated on polyethyleneimine modified iron oxide nanoparticles (Fe_3_O_4_-PEI-Au); Fe_3_O_4_@Citrate/GCE—glassy carbon electrode modified with citrate coated Fe_3_O_4_; Fe_3_O_4_@PDA@MnO_2_/mGCE—magnetic glassy carbon electrode modified with dense polydopamine (PDA) and MnO_2_ coated Fe_3_O_4_ magnetic nanoparticles; Fe_3_O_4_/GN/GE/GCE—glassy carbon electrode modified with graphene/Fe_3_O_4_ nanosheets functionalized with garlic extract; Fe_3_O_4_/SiO_2_/CS/Nafion/GCE—glassy carbon electrode modified with Nafion and chitosan as well as SiO_2_ coated Fe_3_O_4_ magnetic nanoparticles; Fe_3_O_4_@Au/CA/T-COOH/SPCE—thymine acetic acid anchored with cysteamine-conjugated core shell Fe_3_O_4_@Au nanoparticles (Fe_3_O_4_@Au/CA/T-COOH), immobilized on a sensing area of a screen-printed carbon electrode (SPCE); mCPE/HNTs-Fe_3_O_4_–MnO_2_—magnetic carbon paste electrode modified with composite used for extraction of Hg(II)—halloysite nanotubes-ironoxide–manganese oxide nanocomposite; GCE/GO-g-Fe_3_O_4_-PAMA-COOH/AuNPs—glassy carbon electrode modified with dendrimer and Fe_3_O_4_ modified magnetic graphene oxide (GO); Au NPs/Fe_3_O_4_/GCE—glassy carbon electrode modified with Fe_3_O_4_ magnetic nanoparticles and Au nanoparticles; mCPE/MNPZ/Cr(III)/ or mCPE/MNPZ/Cr(VI)—Cr (III/IV) accumulated magnetic carbon paste electrode modified with Fe_3_O_4_ magnetic nanoparticles modified by ZrO_2_ (MNPZs). * The units of analytical parameters presented in the publication have been converted to nmol L^−1^.

**Table 2:** Selected examples of voltammetric procedures for the simultaneous determination of two HMIs using sensors based on Fe_3_O_4_ magnetic nanoparticles.

Sensor	Technique	Base Electrolyte Composition	Analytical Parameters [mol L^−1^]	Determined Ions [mol L^−1^]		Application	Ref.
Simultaneous determination of Cd(II) and Pb(II):				Cd(II)	Pb(II)		
Fe_3_O_4_@G_2_-PAD/mCPE	SWASV	0.1 mol L^−1^ CH_3_COOH/CH_3_COONa pH = 5.5	LOD * Linear range *	1.87 × 10^−9^ 4.45 × 10^−9^–7.11× 10^−7^	8.2 × 10^−10^ 2.41 × 10^−9^–3.86 × 10^−7^	river water, wastewater, and lake water	[40]
GSH@Fe_3_O_4_/mGCE	SWASV	0.1 mol L^−1^ CH_3_COOH/CH_3_COONa pH = 4.5	LOD * Linear range *	1.53 × 10^−9^ 4.49 × 10^−9^–8.90 × 10^−7^	8.7 × 10^−10^ 2.41 × 10^−9^–4.82 × 10^−7^	water samples	[5]
Fe_3_O_4_/Bi_2_O_3_/C_3_N_4_/GCE	SWASV	0.1 mol L^−1^ CH_3_COOH/CH_3_COONa pH = 5.0	LOD Linear range	3.00 × 10^−9^ 1.00 × 10^−8^–3.0 × 10^−7^	1.00 × 10^−9^ 1.00 × 10^−8^–3.0 × 10^−7^	river water	[33]
BiF/Fe_3_O_4_/MWCNTs/LSG/CS/GCE	SWASV	0.1 mol L^−1^ CH_3_COOH/CH_3_COONa pH = 5.0	LOD * Linear range *	8.90 × 10^−10^ 8.90 × 10^−9^–1.78 × 10^−6^	3.40 × 10^−10^ 4.83 × 10^−9^–9.65 × 10^−7^	tap water	[26]
GO-Fe_3_O_4_-PAMAM/GCE	SWASV	0.1 mol L^−1^ CH_3_COOH/CH_3_COONa pH = 4.5 + 0.1 mol L^−1^ KCl	LOD * Linear range *	6.20 × 10^−10^ 1.78 × 10^−9^–1.25 × 10^−6^	6.30 × 10^−10^ 1.93 × 10^−9^–5.79 × 10^−7^	lake and river samples	[41]
PDA@Fe_3_O_4_/mGCE	SWASV	0.1 mol L^−1^ CH_3_COOH/CH_3_COONa pH = 5.0	LOD Linear range	9.20 × 10^−11^ 2.0 × 10^−8^–5.9 × 10^−7^	1.40 × 10^−11^ 5.0 × 10^−9^–6.0 × 10^−7^	aqueous effluent	[67]
GCE/ Nafion@MWCNTs@PEG-Fe_3_O_4_/BiF	DPASV	0.075 mol L^−1^ CH_3_COOH/CH_3_COONa pH = 4.5 + 2.75 μmol L^−1^ Bi(III)	LOD Linear range	4.90 × 10^−10^ 2.0 × 10^−9^–5.0 × 10^−7^	4.60 × 10^−10^ 2.0 × 10^−9^–5.0 × 10^−7^	SRM 1640a, Tap water, spring water, and mineral water	[52]
Simultaneous determination of Hg(II) and Pb(II):				Hg(II)	Pb(II)		
Fe_3_O_4_@SiO_2_-NH_2_/mGCE	DPASV	1.0 mol L^−1^ CH_3_COOH/CH_3_COONa pH = 5.0	LOD Linear range	9.09 × 10^−9^ 3.0 × 10^−8^–5.0 × 10^−5^	6.06 × 10^−9^ 2.0 × 10^−8^–1.0 × 10^−4^	milk	[63]
Simultaneous determination of Hg(II) and Ag(I):				Hg(II)	Ag(I)		
DNA Modified Fe_3_O_4_@AuNPs/mGCE	SWV	Tris-HCl pH 7.4 + 0.14 mol L^−1^ NaCl and 0.005 MgCl_2_	LOD Linear range	1.70 × 10^−9^ 1.0 × 10^−8^–1.0 × 10^−7^	3.40 × 10^−9^ 1.0 × 10^−8^–1.5 × 10^−7^	lake water, drinking water, orange juice, and red wine	[45]
Simultaneous determination of As(III) and Cu(II):				Cd(II)	As(III)		
GCE/GO/Fe_3_O_4_@PMDA/AuNPs	SWASV	0.1 mol L^−1^ CH_3_COOH/CH_3_COONa pH = 6.0 + 0.1 mol L^−1^ KCl	LOD * Linear range *	1.73 × 10^−9^ 7.87 × 10^−9^–1.18 × 10^−5^	2.00 × 10^−9^ 6.67 × 10^−8^–6.67 × 10^−6^	drinking water, pool water, and agricultural water pools	[42]

**Techniques:** SWASV—square-wave anodic stripping voltammetry; DPASV—differential-pulse anodic stripping voltammetry; SWV—square-wave voltammetry. **Sensors:** Fe_3_O_4_@G_2_-PAD/mCPE—magnetic carbon paste electrode modified with second-generation polyamidoamine dendrimer functionalized Fe_3_O_4_ magnetic nanoparticles; GSH@Fe_3_O_4_/mGCE—magnetic glassy carbon electrode modified with glutathione-functionalized Fe_3_O_4_ magnetic nanoparticles; Fe_3_O_4_/Bi_2_O_3_/C_3_N_4_/GCE—glassy carbon electrode modified with a composite of carbon nitride; BiF/Fe_3_O_4_/MWCNTs/LSG/CS/GCE—Fe_3_O_4_, multi-walled carbon nanotubes, laser scribed graphene, and chitosan composite modified glassy carbon electrode doped with bismuth film; GO-Fe_3_O_4_-PAMAM/GCE—glassy carbon electrode modified with poly(amidoamine) dendrimer functionalized magnetic graphene oxide; PDA@Fe_3_O_4_/mGCE—magnetic glassy carbon electrode modified with polydopamine coated Fe_3_O_4_ magnetic nanoparticles; GCE/ Nafion@MWCNTs@PEG-Fe_3_O_4_/BiF—glassy carbon electrode modified with composite based on Nafion, MWCNTs, PEG-functionalized Fe_3_O_4_, and electrochemically coated with Bi film; Fe_3_O_4_@SiO_2_-NH_2_/mGCE—magnetic carbon electrode modified with amino-functionalized Fe_3_O_4_ magnetic nanoparticles; DNA Modified Fe_3_O_4_@Au NPs/mGCE—magnetic glassy carbon electrode modified with DNA modified Au coated Fe_3_O_4_ magnetic nanoparticles; GCE/GO/Fe_3_O_4_@PMDA/AuNPs—glassy carbon electrode modified with nanocomposite based on poly methyldopa along with gold nanoparticles immobilized on the surface of magnetic graphene oxide. * The units of analytical parameters presented in the publication have been converted to nmol L^−1^.

**Table 3:** Selected examples of voltammetric procedures for the determination of multiple HMIs using sensors based on Fe_3_O_4_ magnetic nanoparticles.

Sensor	Technique	Base Electrolyte Composition	Determined Ions	Analytical Parameters [mol L^−1^]		Application	Ref.
Simultaneous determination of Cd(II), Pb(II) and As (III):				LOD *	Linear range *		
(BiO)_2_CO_3_-rGO-Nafion/SPE + Fe_3_O_4_-Au-IL/SPE	SWASV	0.2 mol L^−1^ CH_3_COOH/CH_3_COONa pH = 5.0	Cd(II) Pb(II) As(III)	7.12× 10^−9^ 5.79 × 10^−9^ 3.20 × 10^−8^	0–4.45 × 10^−7^ 0–2.41 × 10^−7^ 0–6.67 × 10^−7^	Simulated river water	[69]
Simultaneous determination of Cd(II), Pb(II) and Hg(II):				LOD	Linear range		
SPE/MBA-BiFE	SWASV	0.1 mol L^−1^ CH_3_COOH/CH_3_COONa pH = 4.5	Cd(II) Pb(II) Hg(II)	3.60× 10^−11^ 3.00 × 10^−12^ 1.10 × 10^−11^	1.00 × 10^−10^–3.00 × 10^−6^ 1.00 × 10^−11^–2.50 × 10^−6^ 1.00 × 10^−10^–2.00 × 10^−6^	tap water, lake water	[70]
TA/Fe_3_O_4_ modified GCE	SWASV	0.1 mol L^−1^ CH_3_COOH/CH_3_COONa pH = 5.0	Cd(II) Pb(II) Hg(II)	2.00× 10^−7^ 4.00 × 10^−8^ 3.00 × 10^−7^	4 × 10^−7^–1.10 × 10^−6^ 4.4 × 10^−7^–1.10 × 10^−6^ 4.4 × 10^−7^–1.10 × 10^−6^	river water	[71]
Simultaneous determination of Cd(II), Pb(II), Cu(II) and Hg(II):				LOD	Linear range		
Fe_3_O_4_@SiO_2_/mGCE	DPASV	1.0 mol L^−1^ CH_3_COOH/CH_3_COONa pH = 5.0	Cd(II) Pb(II) Cu(II) Hg(II)	5.61× 10^−8^ 1.65 × 10^−8^ 7.94 × 10^−8^ 5.67 × 10^−8^	1.00 × 10^−7^–1.00 × 10^−4^ 1.00 × 10^−7^–8.00 × 10^−5^ 1.00 × 10^−7^–8.00 × 10^−5^ 1.00 × 10^−7^–1.00 × 10^−4^	milk samples	[68]
F-MWCNTs/Fe_3_O_4_/Nafion/GCE	SWASV	0.1 mol L^−1^ CH_3_COOH/CH_3_COONa pH = 5.0	Cd(II) Pb(II) Cu(II) Hg(II)	5.00× 10^−11^ 8.00 × 10^−11^ 2.00 × 10^−11^ 5.00 × 10^−11^	5.00 × 10^−10^–3.00 × 10^−8^ 5.00 × 10^−10^–3.00 × 10^−8^ 5.00 × 10^−10^–3.00 × 10^−8^ 5.00 × 10^−10^–2.00 × 10^−8^	river water, soybean	[27]
Simultaneous determination of Cd(II), Pb(II), Cu(II), Zn(II) and Hg(II):				LOD	Linear range		
F-MWCNT/Fe_3_O_4_/0.5% Nafion/GCE	SWASV	0.1 mol L^−1^ CH_3_COOH/CH_3_COONa pH = 5.0	Cd(II) Pb(II) Cu(II) Zn(II) Hg(II)	1.40× 10^−8^ 8.40 × 10^−9^ 5.30 × 10^−9^ 1.20 × 10^−8^ 3.90 × 10^−9^	4.80 × 10^−8^–3.00 × 10^−5^ 2.80 × 10^−8^–3.00 × 10^−5^ 1.70 × 10^−8^–3.15 × 10^−5^ 3.90 × 10^−8^–3.25 × 10^−5^ 1.30 × 10^−8^–3.25 × 10^−5^	lake water, laboratory water, and rice samples	[36]
Individual determination of Cd(II), Pb(II) and Cu(II):				LOD	Linear range		
Fe_3_O_4_@C/GCE	SWASV	0.1 mol L^−1^ CH_3_COOH/CH_3_COONa pH = 5.0	Cd(II) Pb(II) Cu(II)	4.09 × 10^−8^ 2.07 × 10^−8^ 7.93 × 10^−8^	5.00 × 10^−7^–1.30 × 10^−5^ 1.00 × 10^−6^–9.00 × 10^−6^ 4.00 × 10^−7^–9.40 × 10^−6^	-	[57]
NH_2_-Fe_3_O_4_@C/GCE	SWASV	0.1 mol L^−1^ CH_3_COOH/CH_3_COONa pH = 5.0	Cd(II) Pb(II) Cu(II)	2.31 × 10^−8^ 2.85 × 10^−8^ 3.84 × 10^−8^	6.00 × 10^−7^–9.00 × 10^−6^ 1.20 × 10^−6^–1.00 × 10^−5^ 4.00 × 10^−7^–9.40 × 10^−6^	tap water	[57]
Individual determination of Cd(II), Pb(II), Cu(II) and Hg(II):				LOD	Linear range		
Fe_3_O_4_-chitosan NPs/GCE	SWASV	0.1 mol L^−1^ CH_3_COOH/CH_3_COONa pH = 5.0	Cd(II) Pb(II) Cu(II) Hg(II)	3.92× 10^−8^ 4.22 × 10^−8^ 9.67 × 10^−8^ 9.57 × 10^−8^	1.20 × 10^−6^–1.70 × 10^−6^ 1.00 × 10^−7^–1.30 × 10^−6^ 3.00 × 10^−7^–1.20 × 10^−6^ 4.00 × 10^−7^–1.10 × 10^−6^	river water Pb(II) content	[38]
Fe_3_O_4_/GCE	SWASV	0.1 mol L^−1^ CH_3_COOH/CH_3_COONa pH = 5.0	Cd(II) Pb(II) Cu(II) Hg(II)	1.54 × 10^−7^ 1.19 × 10^−7^ 7.65 × 10^−8^ 8.39 × 10^−8^	3.00 × 10^−7^–1.30 × 10^−6^ 3.00 × 10^−7^–1.30 × 10^−6^ 3.00 × 10^−7^–1.70 × 10^−6^ 1.30 × 10^−6^–1.80 × 10^−6^	river water	[64]

**Techniques:** SWASV—square-wave anodic stripping voltammetry; DPASV—differential-pulse anodic stripping voltammetry. **Sensors:** (BiO)_2_CO_3_-rGO-Nafion/SPE—screen-printed electrode modified with Nafion and (BiO)_2_CO_3_-reduced graphene oxide; Fe_3_O_4_-Au-IL/SPE—screen-printed electrode modified with Au modified Fe_3_O_4_ magnetic nanoparticles and ionic liquid; SPE/MBA-BiFE—magnetic Fe_3_O_4_ nanoparticle-decorated phosphorus-doped biochar-ATP/bismuth film screen-printed electrode; Fe_3_O_4_@SiO_2_/mGCE—magnetic carbon electrode coated with TEOS modified Fe_3_O_4_ magnetic nanoparticles; F-MWCNTs/Fe_3_O_4_/Nafion/GCE—glassy carbon electrode modified with Nafion, Fe_3_O_4_ nanoparticles and multi-walled carbon nanotubes (MWCNTs); Fe_3_O_4_@C/GCE—glassy carbon electrode modified with Fe_3_O_4_/carbon magnetic microspheres; NH_2_-Fe_3_O_4_@C/GCE Fe_3_O_4_-- glassy carbon electrode modified with NH_2_ functionalized Fe_3_O_4_/carbon magnetic microspheres; chitosan NPs/GCE—glassy carbon electrode modified with chitosan coated Fe_3_O_4_; Fe_3_O_4_/GCE—glassy carbon electrode modified with Fe_3_O_4_ magnetic nanoparticles. * The units of analytical parameters presented in the publication have been converted to nmol L^−1^.

## 4. Conclusions and Perspectives

Magnetic nanoparticles of iron oxide are gaining popularity due to their excellent physicochemical properties, as well as their very good adsorption and catalytic properties towards HMIs. They are used in many industries and are becoming a desirable electrode material in voltammetry [5,21,26,27,28,29]. There are numerous methods for synthesizing magnetite nanoparticles. Among them, hydrothermal and co-precipitation methods are the most commonly employed in laboratory practice. The former offers advantages such as better control over nanoparticle shape and size distribution, while the latter is simpler, may be conducted under atmospheric pressure and lower temperature, and is more readily scalable [48]. A significant drawback of Fe_3_O_4_ MNPs is their tendency to oxidize and aggregate [26,33]. To mitigate these issues, they are often coated with a suitable organic or inorganic material. This coating not only reduces oxidation and aggregation but also introduces additional active sites capable of interacting with the analyte. Among the various methods for this modification, encapsulation is one of the most effective [48,49]. Another approach involves using a carbon-based material as a base for the nanoparticles. This strategy increases the number of active sites, reduces nanoparticle aggregation, and improves conductivity [26,27]. Consequently, this leads to very good analytical parameters (very low LODs, wide linear ranges of calibration curves—see Table 1, Table 2 and Table 3) for voltammetric procedures used in the determination of HMIs. Moreover, mentioned procedures are characterised by sensors stable for a long time (even ten weeks, with 13.86% signal loss compared to the initial signal) and very good repeatability (RSD < 6.3%) [24,26,27,41,68].

The prepared electrode materials described in the literature were applied onto GCE, mGCE, mCPE, and SPE electrodes, with the first two being the most popular (see Table 1, Table 2 and Table 3). The prepared ink was applied after synthesis onto the electrode surface before measurements [23] or was first used for the extraction of HMIs [66]. The sensors were usually dried using air, under an infrared lamp, or in a laboratory dryer before measurement. This process consolidates the applied composite layer but also improves the stability of the electrodes over time [52].

The developed procedures allow for quantitative analysis of many HMIs such as Ag(I), Cd(II), Pb(II), Hg(II), Cu(II), Zn(II), As(III), and Cr(III/IV), using sensors based on Fe_3_O_4_ magnetic nanoparticles (see Table 1, Table 2 and Table 3). They allow for determination of single ions such as Ag(I) [37], Cd(II) [62], or As(III) [9], as well as the analysis of multiple ions at the same time, e.g., Cd(II), Pb(II), Cu(II), Zn(II), and Hg(II) [36]. However, it should be noted that multi-element determination procedures are burdened with many problems, such as the competitive deposition of the determined ions, the creation of intermetallic compounds, an insufficient number of active sites, and the risk of sensor clogging [27,68]. Measurements are usually performed in an electrolyte with an acidic pH directly using the SWASV [40] and DPASV [60] techniques, as well as using CV and EIS [7]. The developed procedures exhibit very good analytical parameters, including wide linearity ranges for calibration curves and low detection limits. They have been successfully applied for the trace analysis of various metal ions in both aqueous and solid samples (see Table 1, Table 2 and Table 3).

Current trends clearly indicate the dynamic utilization of Fe_3_O_4_ nanoparticles in science, especially in electrochemistry. Their straightforward synthesis and ease of modification make them a very versatile material to work with. It is expected that, in the coming years, the interest in iron oxide nanoparticles will persist. Researchers will be aiming for greater automation of their procedures and multi-element analysis, though challenging, will become an increasingly popular.

## Data Availability

New data were not created or analysed in this study. Data sharing is not applicable to this paper.
